# Comparative genomic analysis of *Acinetobacter* strains isolated from murine colonic crypts

**DOI:** 10.1186/s12864-017-3925-x

**Published:** 2017-07-11

**Authors:** Azadeh Saffarian, Marie Touchon, Céline Mulet, Régis Tournebize, Virginie Passet, Sylvain Brisse, Eduardo P. C. Rocha, Philippe J. Sansonetti, Thierry Pédron

**Affiliations:** 1 Unité de Pathogénie Microbienne Moléculaire, INSERM U1202, Institut Pasteur, Paris, France; 2 Unité de Génomique Evolutive des Microbes, CNRS, UMR3525, Institut Pasteur, Paris, France; 3 Unité de Pathogénie Microbienne Moléculaire, INSERM U1202, Imagopole Citech, Institut Pasteur, Paris, France; 4 Chaire de Microbiologie et Maladies Infectieuses, Collège de France, Paris, France

**Keywords:** *Acinetobacter*, Murine intestine, Comparative genomics, Xenobiotics

## Abstract

**Background:**

A restricted set of aerobic bacteria dominated by the *Acinetobacter* genus was identified in murine intestinal colonic crypts. The vicinity of such bacteria with intestinal stem cells could indicate that they protect the crypt against cytotoxic and genotoxic signals. Genome analyses of these bacteria were performed to better appreciate their biodegradative capacities.

**Results:**

Two taxonomically different clusters of *Acinetobacter* were isolated from murine proximal colonic crypts, one was identified as *A. modestus* and the other as *A. radioresistens*. Their identification was performed through biochemical parameters and housekeeping gene sequencing. After selection of one strain of each cluster (*A. modestus* CM11G and *A. radioresistens* CM38.2), comparative genomic analysis was performed on whole-genome sequencing data. The antibiotic resistance pattern of these two strains is different, in line with the many genes involved in resistance to heavy metals identified in both genomes. Moreover whereas the operon *benABCDE* involved in benzoate metabolism is encoded by the two genomes, the operon *antABC* encoding the anthranilate dioxygenase, and the phenol hydroxylase gene cluster are absent in the *A. modestus* genomic sequence, indicating that the two strains have different capacities to metabolize xenobiotics. A common feature of the two strains is the presence of a type IV pili system, and the presence of genes encoding proteins pertaining to secretion systems such as Type I and Type II secretion systems.

**Conclusions:**

Our comparative genomic analysis revealed that different *Acinetobacter* isolated from the same biological niche, even if they share a large majority of genes, possess unique features that could play a specific role in the protection of the intestinal crypt.

**Electronic supplementary material:**

The online version of this article (doi:10.1186/s12864-017-3925-x) contains supplementary material, which is available to authorized users.

## Background

*Acinetobacter,* belonging to γ-proteobacteria, are gram-negative strictly aerobic, non-motile, non-fermentative and oxidase-negative bacteria. The classification of the genus *Acinetobacter* contains over 50 species [1]. *Acinetobacter* spp. were considered for decades as saprophytic environmental microorganisms. However, recently, they have increasingly been implicated in various types of infections, mainly nosocomial infections in fragilized patients in intensive care units, hence adding the hospital to the list of their favorite environments. A major trait of their pathogenicity is their high and broad array of antibiotic resistance. The often multi-drug resistance (MDR) *Acinetobacter baumannii* is the major species in the genus involved in recent nosocomial infections. In contrast to *A. baumannii* that is mainly found in the hospital environment, other species of the *Acinetobacter* genus are isolated from the soil, water, and animals [2]. Many environmental *Acinetobacter* spp. are able to metabolize pollutants such as “*Acinetobacter oleivorans”* DR1 that degrades diesel [3] and the strains *Acinetobacter pittii* PHEA-2 and *Acinetobacter baylyi* ADP1 that degrade phenol [4].

Our previous data showed the existence of a Crypt Specific Core Microbiota (CSCM) in the caecum and proximal colon of laboratory mice of various lineages, and identified a restricted set of strictly aerobic, non-fermentative bacterial genus, dominated by members of the *Acinetobacter* genus showing unexpected tropism for the crypt environment [5]. It was shown that oxygen is present at the gut mucosal surface at low but significant concentration thereby facilitating the growth of strictly aerobic and aero-anaerobic bacteria, allowing them to be biochemically active [6]. We hypothesize that these bacteria act as a crypt “gate keeper” by protecting the crypt regenerative apparatus, particularly stem cells, against cytotoxic and genotoxic signals. This may occur by several means: by preventing colonization by pathobionts, by regulating local innate immune mechanisms to avoid chronic low grade inflammation, and by providing strong biodegradative capacities against xenobiotics leading to putative cancer protective effects. Indeed as these bacteria are in close vicinity of intestinal stem cells we hypothesize that they act in order to keep homeostasis in this particular niche and also participate to the control of intestinal proliferation. The aim of the present study was to characterize and compare the genome of two different strains of *Acinetobacter* isolated from murine colonic crypts. We show here that these two strains belong to two different species of *Acinetobacter* found in the crypts (*Acinetobacter modestus* and *Acinetobacter radioresistens*), harbor different patterns of antibiotic resistance and also possess different xenobiotic degradative properties. It is interesting to notice, for instance, that efficient dehalogenation of xenobiotics requires aerobic conditions and that the CSCM are aerobic bacteria.

## Methods

### Isolation of crypt specific core Acinetobacter from murine proximal colon

Proximal colonic tissues from C57Bl/6 mice (Elevage Janvier) were washed with bleach and homogenized using in 2 ml of sterile PBS using the Precellys system with 2,8 mm ceramic beads and added to 30 ml of a minimum medium [7]. The cultures were incubated at 30 °C during 48H under shaking condition (300 rpm). The cultures were then isolated on agar plates (GTCS, MacConkey, Herellea, ChromAgar). Selected colonies were then re-isolated on Chromagar plates. Bacteria were identified using the Biolog system (GEN III MicroPlate for both Gram-negative & Gram-positive bacteria, 21,124 Cabot Blvd. Hayward CA, 94,545 USA). The identification of *Acinetobacter* was confirmed by Sanger sequencing of *16S rDNA*, and recombinase A (*recA*) after genomic DNA extraction using the Wizard Genomic DNA Purification Kit following manufacture’s instructions (Promega) and PCR amplification. The primers used are listed in the Additional file 1: Table S1.

### Antimicrobial susceptibility testing

Antibiotic susceptibility was determined by the disk diffusion method on Mueller-Hinton (MH) agar according to the guidelines of the Antibiogram Committee of the French Microbiology Society [8]. Automatic readings were performed using the OSIRIS system (Bio-Rad).

### Biofilm assay

Biofilm formation was determined using an overnight culture, diluted 1:100 in fresh Trypticase-Soy broth in 96-well polystyrene plates and incubated without shaking at 37 °C for 24 h and 48 h. After incubation the plates were washed gently three times with phosphate-buffered saline to remove unattached bacteria, air-dried and stained with 0.1% crystal violet solution for 20 min and quantified at 595 nm after solubilization with ethanol–acetone [9]. Of the 96 wells, six were left uninoculated and used as background controls. *Escherichia coli* DH5α and *Acinetobacter baumannii* CIP 70.34^T^ (ATCC 19606) were used respectively as negative and positive control of biofilm formation [10]. The biofilm formation experiments were carried out with six replicates and the results are expressed as mean values of crystal violet absorbance ± SD from the mean.

### Genome sequencing, assembling, annotation

The genome sequencing, assembling and annotations of *A. modestus* CM11G and *A. radioresistens* CM38.2 obtained through Illumina paired-end sequencing were already described in [11]. The complete genome sequence of *A. radioresistens* CM38.2 was obtained using PacBio single-molecule real-time (SMRT) technology [12] with P6-C4 chemistry in the PacBio RS II sequencing platform (https://www.gatc-biotech.com). One SMRT cell generated 72,134 reads with a mean read length of 15,211 bp. The reads were assembled de novo with the Hierarchical Genome Assembly Process 3 (HGAP3) [13] giving one contig of 3,201,807 bp with a G + C % of 41.7 and with an average coverage depth of 275. Whole-genome alignment of the *A. radioresistens* CM38.2 strain was performed using Mauve v 2.31 software [14] in order to compare the sequences obtained either by PacBio or by Illumina paired-end sequencing (Additional file 2: Figure S1).

The complete sequence of CM38.2 and all 118 contigs described in [11] of CM11G were annotated using the RAST and MicroScope platforms [15–17]. For both platforms our annotation job were submitted by providing mandatory information and accepting default parameters. Both platforms give access to several tools of visualization and comparative genomics and produced very similar results. In order to homogenize the results, we used only the annotations obtained with RAST.

### Core-genomes

The core-genome of the species is defined as the intersection of pairwise lists of strict positional orthologs. We built three core-genomes: i) containing the 133 *Acinetobacter* strains used in [18] plus our two strains CM11G, CM38.2, ii) for the species *A. radioresistens,* iii) for *A. modestus*. All three core-genomes were built following the same protocol (as detailed in [19]). Briefly, orthologs were identified as bidirectional best hits using end-gap free global alignment, between the proteome of *A. baumannii* AYE as a pivot and each of the other proteomes (135 for the genus and 3 for the two species). Hits with less than 40% (genus) or 80% (species) similarity in amino acid sequence or more than 20% difference in protein length were discarded. Genomes from the same species typically show low levels of genome rearrangements and this information can be used to identify orthologs more accurately [20, 21]. The core-genomes consist in the genes present in all genomes of each of the three sets.

Average nucleotide identity (ANIb) was computed using JSpecies v.1.2.1 [http://imedea.uib-csic.es/jspecies/] based on BLAST+ (v.2.2.29). The ANIb values were thus generated, on the one hand for pair-wise comparisons of CM38.2 and *A. radioresistens* strains NIPH 2130 (accession number NZ_APQE00000000.1) and CIP 103788 (accession number NZ_APQF00000000.1), and on the other hand for the comparison of CM11G with *A. modestus* strains ANC 3862 (accession number NZ_APRP00000000.1) and NIPH 236 (accession number NZ_APOJ00000000.1).

### Pan-genomes

The pan-genome of the species is defined as the union of all the homologs present in the genes set of all strains, and provides information regarding the genetic diversity of the set of genomes. The pan-genomes were built for the same three sets of strains previously described (see Core-genomes section). In each case, the pan-genome was obtained by clustering homologous proteins into families. The lists of putative homologs between pairs of genomes using “all-against-all” comparisons with BLASTp v.2.2.28+ (default parameters) [22] were determined and then clustered by similarity using Silix v1.2.8 (http://lbbe.univ-lyon1.fr/SiLiX) [23] when the e-values were smaller than 10^−4^. A protein is thus included in the family if it shares a relation of homology to a protein already in the family. Silix parameters were set such that a protein was homologous to another in a given family if the alignment had at least 40% (genus) or 80% (species) sequence identity and included more than 80% of the smallest protein.

### Phylogenetic analyses

For each of the phylogenetic reconstructions, we used the model minimizing the Bayesian Information Criterion (BIC) among all models available (option -m TEST) in IQ-TREE. We made 1000 ultra fast bootstraps to evaluate node support (options –bb 1000 –wbtl in IQ-TREE).

#### 16S rRNA gene phylogenetic tree

We built a tree to display the phylogenetic distribution of our dataset using the 16S rRNA genes sequences of the 133 *Acinetobacter* strains used in [18] plus our 10 additional strains. We made a multiple alignment of the 16S rRNA sequences with INFERNAL v.1.1 (default parameters) [24] using RF00177 Rfam model (v.12.1, [25]), followed by manual correction with SEAVIEW to remove poorly aligned regions. The tree was computed by maximum likelihood with IQ-TREE multicore v.1.4.2 [26] under the TVM + I + G4 model.

#### *recA* gene phylogenetic tree

We extracted from the genus core-genome the gene family encoding RecA. Then we made multiple alignment of the set including the 135 *recA* DNA sequences and our eight additional strains with MAFFT v.7.205 (default parameters) [27], followed by manual correction with SEAVIEW to remove poorly aligned regions. The phylogenetic tree was inferred using IQ-TREE multicore v.1.4.2 under the TIM3 + I + G4 model.

#### Core-genome phylogenetic tree

Each of the 945 families of proteins of the *Acinetobacter* core-genome was used to produce a multiple alignment with MAFFT v.7.205 (default parameters) [27]. Poorly aligned regions were removed with BMGE (default parameters) [28]. The phylogenetic tree was inferred using IQ-TREE multicore v.1.4.2 under the LG + I + G4 + F model.

## Results

### Isolation and characterization of *Acinetobacter* strains in murine colonic crypts

As *Acinetobacter* was the major genus found in murine proximal colonic crypts, a selective minimum medium was used in order to isolate these strains [7, 11]. Among the different preparations, ten *Acinetobacter* strains were isolated on ChromAgar plates and identified using the Biolog System based on 71 carbon source utilization assays and 23 chemical sensitivity assays. Eight of the strains were identified as *Acinetobacter* genospecies 6 and the two others as *A. radioresistens* indicating a clear separation of the ten strains into two clusters (Table 1).

**Table 1:** Biochemical identification of the isolated strains

Strain	Biolog Identification
CM11G	*Acinetobacter genospecies 6*
CM31.3	*Acinetobacter genospecies 6*
CM31.5	*Acinetobacter genospecies 6*
CM31.6	*Acinetobacter genospecies 6*
CM32.1_HC	*Acinetobacter genospecies 6*
CM32.1	*Acinetobacter genospecies 6*
CM37.1	*Acinetobacter genospecies 6*
CM37.2	*Acinetobacter genospecies 6*
CM38.1	*Acinetobacter radioresistens*
CM38.2	*Acinetobacter radioresistens*

10 *Acinetobacter* isolates were identified by the Biolog System using GEN III MicroPlate based on 71 carbon source utilization assays and 23 chemical sensitivity assays

### Phylogeny of 16S ribosomal RNA and recombinase a genes

**Fig. 1:**
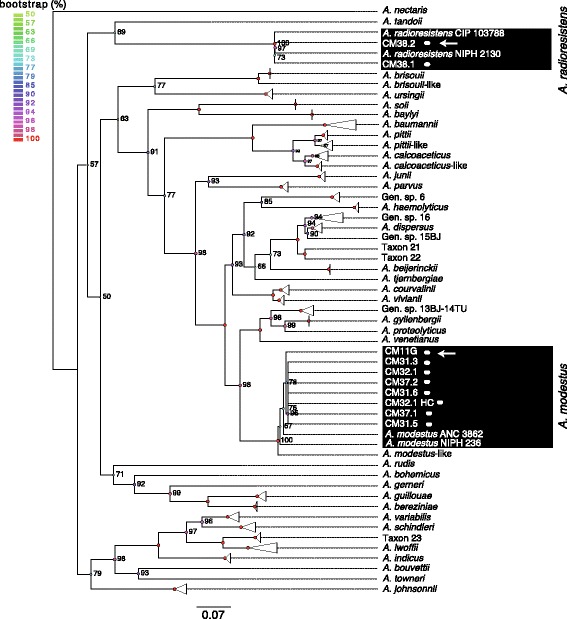
Phylogenetic tree of *Acinetobacter* strains based on *recA* gene sequences. *Triangles* mark groups of taxa that are from the same species. The scale bar represents the average number of substitutions per site

In order to get a more precise identification of the strains, a phylogenetic tree based on 16S ribosomal RNA sequences was built. This phylogenetic tree confirmed the biochemical identification of strains CM38.1 and CM38.2 as closely related to *A. radioresistens*, and indicated that the eight other strains previously identified as *Acinetobacter* genospecies 6 were closely related to *A. modestus* species [29] (Additional file 3: Figure S2). In order to confirm these results we built a phylogenetic tree based on the *recA* sequences because this gene was used to identify the different genospecies of the genus *Acinetobacter* [30]. This tree positioned these eight strains in the vicinity of the *A. modestus* species with more than 99% of sequence similarity and the two strains CM38.1 and CM38.2 in the vicinity of *A. radioresistens* (Fig. 1).

### Whole-genome sequencing

One strain of each cluster was selected for sequencing: - CM11G for *A. modestus* and CM38.2 for *A. radioresistens* - with the Illumina HiSeq 2000 technology (paired-end libraries) [11]. We also sequenced *A. radioresistens* CM38.2 strain (accession number SRR5351953) using the PacBio technology. Annotation with RAST gives 2968 coding DNA sequences (CDS) and 3104 CDS for *A. radioresistens* CM38.2 paired-end and PacBio respectively. This latter annotation was used for the genomic comparative analysis. The distribution of the genes of CM11G and CM38.2 is listed in the Additional file 4: Table S2 according to the functional categories given by RAST.

### *Acinetobacter* Core-genomes

**Fig. 2:**
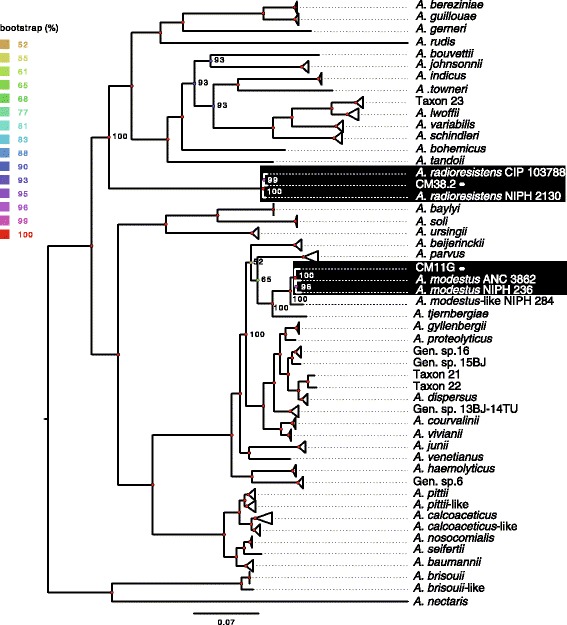
Phylogeny of the *Acinetobacter* genus based on the alignment of the protein families of the core-genome. *Triangles* mark groups of taxa that are from the same species. The scale bar represents the average number of substitutions per site

In order to get an idea of the core-genome based on the sequences of *A. modestus* CM11G and *A. radioresistens* CM38.2, a core-genome of 133 *Acinetobacter* strains [18] plus our two strains CM11G and CM38.2 was built (Table 2). The core-genome of the 135 genomes of *Acinetobacter* consists of 945 families of homologous proteins*.* A phylogenetic tree based on these proteins confirmed the identification of the two strains, since *A. radioresistens* CM38.2 is included is the *A. radioresistens* clade, and CM11G in the *A. modestus* clade (Fig. 2 and Additional file 5: Figure S3). The average nucleotide identity (ANIb) allows to putatively classify bacterial strains in the same species. It is currently admitted that an ANIb value of more than 95% is strong indication that strains belong to the same species [31]. The values of ANIb between CM11G and two *A. modestus* strains (ANC 3862, NIPH 236) are higher than 96%. The values of ANIb for the comparisons between CM38.2 and two *A. radioresistens* strains (NIPH 2130, CIP 103788) are higher than 98%. These results are consistent with those obtained with the phylogenetic tree based on the *Acinetobacter* core-genome (Table 3), and suggest that the two strains are part of well-defined *Acinetobacter* species.

**Table 2:** Core genome of *Acinetobacter*

	Number of genomes	Average number of gene families	Smallest proteome	Number of core gene families (% of the smallest)
*Acinetobacter*	133^a^	3523	2562	950
*Acinetobacter*	135^b^	3529	2562	945
*Acinetobacter radioresistens*	3^c^	2991	2936	2391 (81%)
*Acinetobacter modestus*	3^d^	3930	3347	2637 (79%)

^a^133 strains described in [15]; ^b^133 strains and CM11G and CM38.2; ^c^CM38.2 + NIPH2130 + CIP103788; ^d^CM11G + ANC3862 + NIPH236. Hits with less than 40% (genus) or 80% (species) similarity in amino acid sequence or more than 20% difference in protein length were discarded

**Table 3:** Average Nucleotide Identity (ANIb) in percent between the *A. radioresistens* strains (A) and between *A. modestus* strains (B)

A. *A. radioresistens* Average nucleotide identity			
	CM38.2	NIPH2130	CIP103788
CM38.2	---	98.06	98.11
NIPH2130	98.26	---	98.30
CIP103788	98.12	98.06	---
B. *A. modestus* Average nucleotide identity			
	ANC3862	NIPH236	CM11G
ANC3862	---	96.80	96.72
NIPH236	96.81	---	96.66
CM11G	96.80	96.75	---

The ANIb based on BLAST+ were performed with the software JSpecies

The core-genomes of species *A. modestus* and *A. radioresistens* were built using the three strains available for each (two published and one sequenced by us, see Methods). They contain 2637 and 2391 of orthologous proteins families, corresponding to 79% and 81% of the genomes of our strains for respectively *A. modestus* and *A. radioresistens* (Table 2).

### *Acinetobacter* pan-genomes

**Fig. 3:**
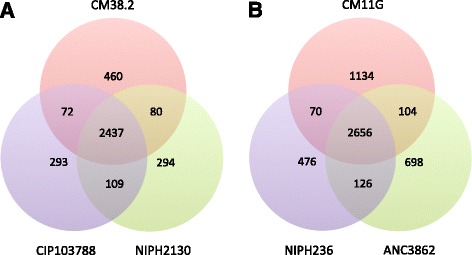
Venn diagrams of the pan-genome of *A. radioresistens* strains (**a**) and *A. modestus* strains (**b**)

The analysis of the core genome showed that both genomes contain many genes absent from the core genome of their species. In order to compare the variability between strains, we analyzed their pan-genomes (see Methods). At the genus level, the 135 *Acinetobacter* strains contained 30,080 protein families (identified using a threshold of 40% similarity, Table 4). At the species level, using a threshold of 80% similarity, we identified 3745 and 5264 proteins for *A. radioresistens* and *A. modestus* respectively (Fig. 3 and Table 4). The genomes of the strains from the crypts were, in both cases, larger than the ones of the other strains of the same species. Accordingly, they have more strain-specific genes than the others, which may provide traits involved in the adaptation to their specific niche.

**Table 4:** Pan genome of *Acinetobacter*

	Number of genomes	40%	50%	70%	80%
*Acinetobacter*	135^a^	30,080	35,274	53,163	71,471
*Acinetobacter radioresistens*	3^b^	3489	3577	3695	3745
*Acinetobacter modestus*	3^c^	4908	5049	5187	5264

^a^133 strains and CM11G and CM38.2; ^b^CM38.2 + NIPH2130 + CIP103788; ^c^CM11G + ANC3862 + NIPH236

### Antibiotic resistance profiles

In order to get a better phenotypic characterization of the ten isolated strains, their antibiotic resistance was analyzed using a panel of 32 antimicrobials agents usually tested for non-fermentative Gram-negative bacteria. As for the biochemical identification and the phylogenetic analysis, the ten strains could be divided into two clusters. The eight *A. modestus* strains were resistant to streptomycin, spectinomycin, and latamoxef (cephalosporin family). The two *A. radioresistens* were sensitive to these antibiotics but resistant to chloramphenicol, unlike the *A. modestus* strains (Additional file 6: Table S3).

Aminoglycoside-modifying enzymes mediate the resistance to aminoglycosides such as streptomycin. A comparative analysis of antibiotic resistance genes performed on the two selected sequenced strains indicated the presence of an O-adenylyltransferase (*aadA*) in the genome of *A. modestus* whereas the gene encoding this enzyme was absent in the genome of *A. radioresistens* CM38.2. This could explain the resistance to streptomycin and spectinomycin of the former strain. *RarD*, encoding a chloramphenicol-sensitive protein, and *cat* genes encoding a chloramphenicol acetyltransferase were present in the two genomic sequences even if *A. modestus* CM11G is sensitive to this antibiotic. Other resistance mechanisms, such as efflux pumps, may contribute to chloramphenicol resistance in *A. radioresistens*, such as the gene *mdfA* coding for a multidrug/chloramphenicol efflux transport belonging to the MFS (major facilitator superfamily) which is present in both strains. The RND (resistance-nodulation-division) family of efflux pumps is often described in genomes of pathogenic MDR strains of *A. baumannii*, mainly the AdeABC system [32]. However these genes were not present in the two sequenced strains analyzed in this study. But other RND type efflux pumps and many genes involved in the resistance to heavy metals such as arsenate, cobalt, zinc, cadmium and also to copper were found in the two genomes (Additional file 7: Table S4).

### Xenobiotic metabolism

**Fig. 4:**
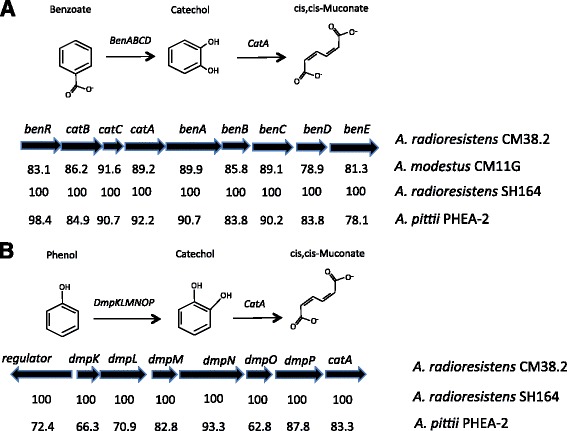
Genetic organization and conservation of the *BenABCDE* cluster (**a**), and of the phenol degradation operon (**b**). The percentage of nucleotides identity between the strains for each gene is indicated

The intestinal microbiota is able to metabolize xenobiotics, including drugs, and thereby modulate their toxicological and pharmacological properties [33]. We wondered if some operons or genes involved in this process were present in our selected strains through the annotation tables. Both *A. modestus* CM11G and *A. radioresistens* CM38.2 carry the operon *benABCDE* encoding benzoate 1,2-dioxygenase alpha (*benA*) and beta (*benB*) subunits with sequence homologies between themselves of 89.9 and 85.8 respectively (Fig. 4A). The operon *antABC* encoding the anthranilate dioxygenase, which catalyzes catechol formation, is present in the genomic sequence of *A. radioresistens* CM38.2, in the available genomic sequence of *A. radioresistens* SH164 (accession number NZ_GG705131), and in the two *A. radioresistens* strains used for the core-genome analysis. In contrast, it is absent in *A. modestus* CM11G. It should be noted that the *antABC* operon is also present in the genome of various *Acinetobacter* species such as *A. baumannii* (AB307 and ATCC 17978), *A. pittii* PHEA-2 (accession number CP002177) and *A. baylyi* ADP1 [34]. Moreover, the phenol hydroxylase gene cluster *dmpKLMNOP*, which convert phenol to catechol, is absent in the genome of *A. modestus* CM11G whereas it is present in *A. radioresistens* CM38.2, *A. radioresistens* SH164 and *A. pittii* PHEA-2 (Fig. 4B) [4]. The organization of phenol degradation genes in these strains indicates a high degree of similarity, with a homology of 100% with *A. radioresistens* SH164 and around 80% with *A. calcoaceticus*. These findings indicate that the strains isolated from murine intestinal crypts are able to metabolize xenobiotics, even if at different levels.

### Secretion systems

A diversity of secretion systems were described in Gram-negative bacteria and in some strains of *Acinetobacter* such a Type I, Type II and Type VI secretion systems that are known to be involved in pathogenicity or in supporting survival in a complex microbial community [35, 36]. The T1SS secreted agglutinin RTX was found in the genomic sequence of *A. radioresistens* CM38.2 and shared a sequence identity of 40% with the biofilm-associated protein (Bap) of *A. baumannii* AB0057. Bap plays a role in the adhesion to host cell and in the maintenance of the biofilm. The gene encoding TolC, a porin at the outer membrane that is part of T1SS and drug-efflux pumps [37], also involved in biofilm formation, is present in the genomic sequences of *A. modestus* CM11G and *A. radioresistens* CM38.2. Quantitative analysis of biofilm formed by these two strains indicates that both *A. modestus* CM11G and *A. radioresistens* CM38.2 are capable to induce biofilm after 24H of incubation even if *A. modestus* CM11G is a stronger inducer of biofilm than *A. radioresistens* CM38.2. After 48H of culture the ability of *A. radioresistens* CM38.2 is weaker, whereas *A. modestus* CM11G remains as strong as *A. baumannii* CIP 70.34^T^ (Additional file 8: Figure S4).

Genes encoding general secretion pathway proteins (gsp) that are associated with the type II secretion system (T2SS) [38] were found in the two strains. The operon encoding the twin-arginine translocation proteins (TatABC), involved in the export of folded proteins across the cytoplasmic membrane of bacteria, also known as sec-independent translocase proteins, was found in the genomic sequences of *A. modestus* CM11G and *A. radioresistens* CM38.2.

A type VI secretion system (T6SS) is present in many *Acinetobacter* isolates, environmental or clinical strains [39] and could be involved in host colonization [40]. This secretion system was not found in the genomic sequences of *A. modestus* CM11G and *A. radioresistens* CM38.2. Interestingly, a T6SS is present in the two *A. radioresistens* strains NIPH 2130 and CIP 103788 suggesting an adaptation of the strain CM38.2 to its niche.

xSeveral genes involved in adhesion and biofilm formation such as type IV pili [41] are represented in both selected strains *A. modestus* CM11G and *A. radioresistens* CM38.2. Even if *Acinetobacter* name means non-motile rod, some species are able of bacterial locomotion through Type IV dependent twitching motility [42]. Many *Acinetobacter* species harbor the genes encoding the proteins necessary for a functional Type IV pili system [43, 44]. Similarly 22 genes among the 24 listed are both present in the genomic sequences of *A. modestus* CM11G and *A. radioresistens* CM38.2 (Table 5). The Type IV pili system is also involved in natural transformation in *Acinetobacter*, where transformation is tightly associated with mobility [45]. Overall, these results indicate that the two selected strains from murine intestinal crypts shared many secretion systems that could allow the colonization of this particular niche.

**Table 5:** Genes involved in Type IV pilus apparatus in the genome of *A. modestus* CM11G and *A. radioresistens* CM38.2

Function	*A. modestus* CM11G	*A. radioresistens* CM38.2	Sequence Identity (%)
3-dehydroquinate synthase (EC 4.2.3.4)	1	1	82.40
Fimbrial assembly protein FimB	1	0	
Leader peptidase (Prepilin peptidase)	1	1	81.12
Multimodular transpeptidase-transglycosylase	1	1	79.35
N-methyltransferase	1	1	81.12
Twitching motility protein PilG	1	1	99.21
Twitching motility protein PilH	1	1	85.83
Twitching motility protein PilT	1	1	57.81
Type IV fimbriae expression regulatory protein PilR	0	1	
Type IV fimbrial assembly protein PilC	1	1	91.67
Type IV fimbrial assembly, ATPase PilB	1	1	80.42
Type IV fimbrial biogenesis protein FimT	1	1	39.88
Type IV fimbrial biogenesis protein PilV	1	1	38.83
Type IV fimbrial biogenesis protein PilW	1	1	36.89
Type IV fimbrial biogenesis protein PilX	1	1	30.53
Type IV fimbrial biogenesis protein PilY1	1	1	30.15
Type IV pili signal transduction protein PilI	1	1	78.65
Type IV pilin PilA	1	1	80.49
Type IV pilus biogenesis protein PilJ	1	1	77.99
Type IV pilus biogenesis protein PilM	1	1	85.81
Type IV pilus biogenesis protein PilN	1	1	78.24
Type IV pilus biogenesis protein PilO	1	1	70.45
Type IV pilus biogenesis protein PilP	1	1	74.57
Type IV pilus biogenesis protein PilQ	1	1	72.78

1: presence of the feature; 0: absence of the feature. The percentage number represents the identity in the sequences of the two genomes

### Siderophores

**Fig. 5:**
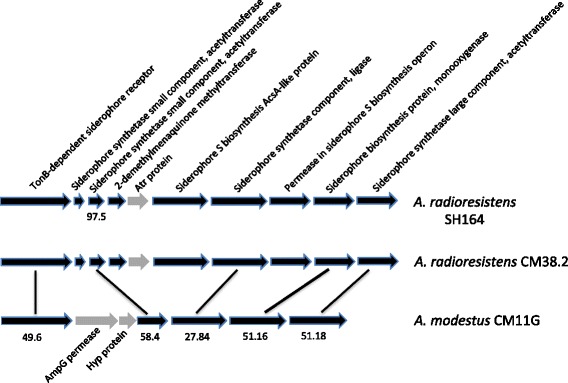
Genetic organization of the siderophore cluster found in *A. radioresistens* CM38.2. The percentage of nucleotide identity with *A. radioresistens* SH164 and *A. modestus* CM11G are indicated

Iron is an essential element for the growth of a large number of bacteria [46]. Several mechanisms to acquire iron have been developed by bacteria, including siderophores. Moreover these siderophores are considered as virulence factors for pathogenic bacteria such as *A. baumannii* that encodes for the highly conserved acinetobactin [47, 48]. A siderophore cluster was found in the genome of *A. radioresistens* CM38.2 whereas some genes were missing in the genome of *A. modestus* CM11G (Fig. 5). Another cluster of four genes is also present in *A. radioresistens* CM38.2 but absent in the *A. modestus* CM11G strain (Additional file 7: Table S4). This cluster encodes proteins involved in the siderophore S biosynthesis. The same clusters for iron uptake are also present in the genome sequence of *A. radioresistens* SH164 with a sequence similarity of 100% except for one of the genes (Fig. 5). This reflects an iron metabolic variability for the two *Acinetobacter* strains.

## Discussion

In this study, we characterized the genomic features of two *Acinetobacter* isolated from murine colonic crypts and belonging to the Crypt Specific Core Microbiota (CSCM). They were isolated from each of the two large clusters of *Acinetobacter* strains that can be distinguished by their biochemical profiles: *A. modestus* and *A. radioresistens*. These two clusters could also be discriminated based on their antibiotic resistance. In contrast to many pathogenic *Acinetobacter* bacteria such as *A. baumannii* that possess a T6SS [40], the T6SS was not encoded by the genome of *A. modestus* and *A. radioresistens*. However, genes encoding proteins involved in Type I secretion system, Type II secretion system and Type IV pili system are present in the genomic sequences of the selected strains. Many others genes involved in heavy metal resistance as well as multidrug resistance efflux pumps are encoded by these strains.

The two *Acinetobacter* shared also some operons such as the *benABCDE* involved in benzoate degradation whereas the operon involved in phenol degradation is absent in the *A. modestus* strain. Does CSCM “gate keeper” exert protection of the epithelial regenerative apparatus against the (geno)toxic potential of metabolic by-products of the microbiota and xenobiotics? Undigested dietary fibers and endogenous residues are metabolized by the gut microbiota and some of the by-products of this microbial metabolism are involved in tumor promotion (secondary bile acids, anaerobic tryptophan degradation products: indoles, ammonia), mutagenesis (fecapenaenes) or oncogenesis (N-nitrosocompounds). Numerous bacterial enzymes responsible for the production of carcinogens have been identified. The protective effect of certain bacterial species is also recognized, encompassing carcinogen binding, detoxification of methylmercury, formation of isoflavones [49]. Regarding xenobiotics, there is a broad range of bacterial dehalogenases that catalyze the cleavage of carbon-halogen bonds, which is a key step in aerobic mineralization pathways of many potentially carcinogenic halogenated compounds that occur as environmental pollutants [50].

This study sheds new light on genomic features involved in xenobiotic metabolism that could play a crucial role in the protection of colonic crypts that harbor the intestinal stem cells.

## Conclusions

In this study, we used whole-genome sequencing to characterize *Acinetobacter* isolated from murine colonic crypts. We performed genomic analysis of two isolates belonging to two different species in comparison to available *Acinetobacter* genomes in public databases. Our results shed new light on genomic features involved in xenobiotic metabolism that could play a crucial role in the protection of colonic crypts that harbor the intestinal stem cells.

## Additional files

Additional file 1: Table S1. Sequence of primers used for identification of the strains based on the sequences of *16S rRNA* and *recA*. (DOCX 33 kb)

Additional file 2: Figure S1. Whole genome comparative alignment of *A. radioresistens* CM38.2. The genome sequence is presented horizontally with the scale showing the sequence coordinates and the conserved shared synteny represented as the colored blocks which are connected across genomes. Upper panel: PacBio sequencing; lower panel: Illumina paired-end sequencing. (PDF 80 kb)

Additional file 3: Figure S2. Complete phylogenetic tree of *Acinetobacter* strains based on *16S rRNA* gene sequences. The scale bar represents the average number of substitutions per site. (PDF 586 kb)

Additional file 4: Table S2. Distribution of the genes of *A. modestus* CM11G and *A. radioresistens* CM38.2 following the functional categories obtained following RAST annotations. (DOCX 73 kb)

Additional file 5: Figure S3. Complete phylogenetic tree of the *Acinetobacter* genus based on the alignment of the protein families of the core-genome. The scale bar represents the average number of substitutions per site. (PDF 302 kb)

Additional file 6: Table S3. Antibiotic resistance pattern of the 10 *Acinetobacter* isolates. S: sensitive; I: intermediate; R: resistant. This panel of 32 antimicrobials agents is usually tested for non-fermentative Gram-negative bacteria. (DOCX 106 kb)

Additional file 7 Table S4. Comparison of the genomes of *A. modestus* CM11G and *A. radioresistens* CM38.2 based on their functional categories according to RAST classification into subsystems. 1: presence of the feature; 0: absence of the feature. (XLSX 97 kb)

Additional file 8: Figure S4. Quantification of biofilm formation. Bacteria were incubated at 37 °C in Trypticase-Soy broth in polystyrene plate for 24H (A) or 48H (B). Data are expressed as mean ± Standard deviation, *n* = 6 in each group. **, *P* < 0.001 versus biofilm formation by *Escherichia coli* DH5a. Control: uninoculated wells. (PDF 56 kb)

## Abbreviations

CSCM: Crypt Specific Core Microbiota;
MDR: multi-drug resistance
recA: recombinase A
RND: resistance-nodulation-division
T6SS: Type VI secretion system
