# Supplementary material for: Comparative genomic analysis of Acinetobacter strains isolated from murine colonic crypts
Source: BMC Genomics. 2017 Jul 11;18:525. doi: 10.1186/s12864-017-3925-x (PMC5505149; doi:10.1186/s12864-017-3925-x)
Supplement: Supplementary file 1 — Sequence of primers used for identification of the strains based on the sequences of 16S rRNA and recA. (DOCX 33 kb) [file 12864_2017_3925_MOESM1_ESM.docx]

| Gene | forward | reverse |
| --- | --- | --- |
| *16S rRNA* | AGAGTTTGATCCTGGCTCAG | GACGGGCGGTGWGTRCA |
| *recA* | CCTGAATCTTCYGGTAAAAC | GTTTCTGGGCTGCCAAACATTAC |
