# Supplementary figures and images for: Comparative genomic analysis of Acinetobacter strains isolated from murine colonic crypts
Source: BMC Genomics. 2017 Jul 11;18:525. doi: 10.1186/s12864-017-3925-x (PMC5505149; doi:10.1186/s12864-017-3925-x)

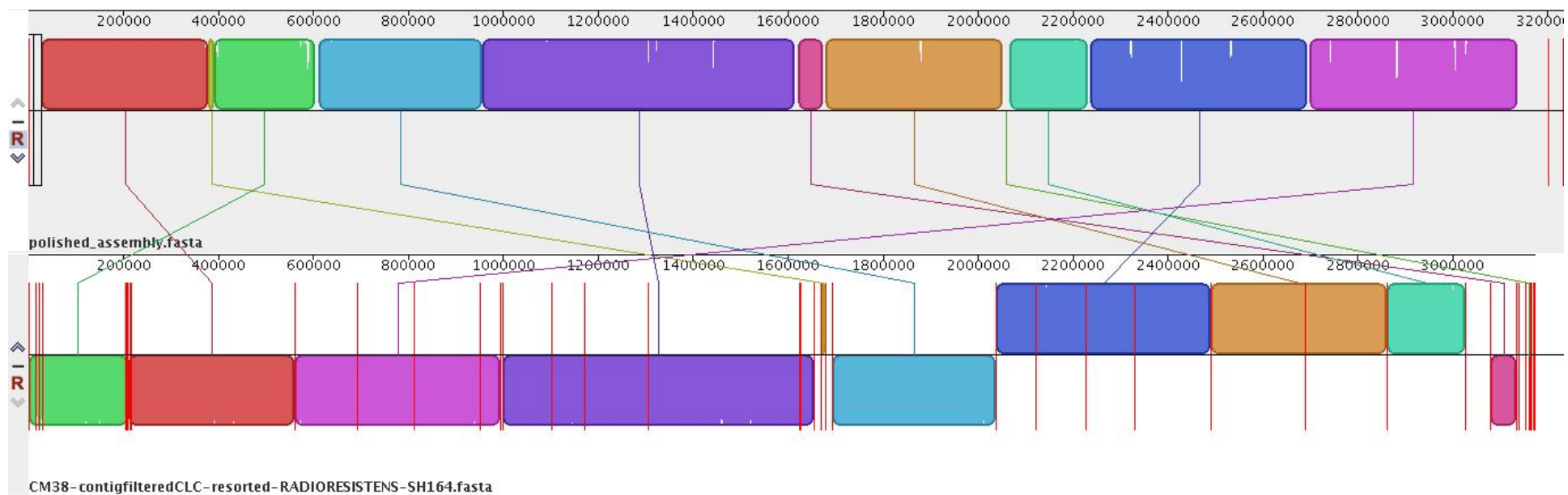

Supplement: Supplementary file 2 — Whole genome comparative alignment of A. radioresistens CM38.2. The genome sequence is presented horizontally with the scale showing the sequence coordinates and the conserved shared synteny represented as the colored blocks which are connected across genomes. Upper panel: PacBio sequencing; lower panel: Illumina paired-end sequencing. (PDF 80 kb) [file 12864_2017_3925_MOESM2_ESM.pdf]

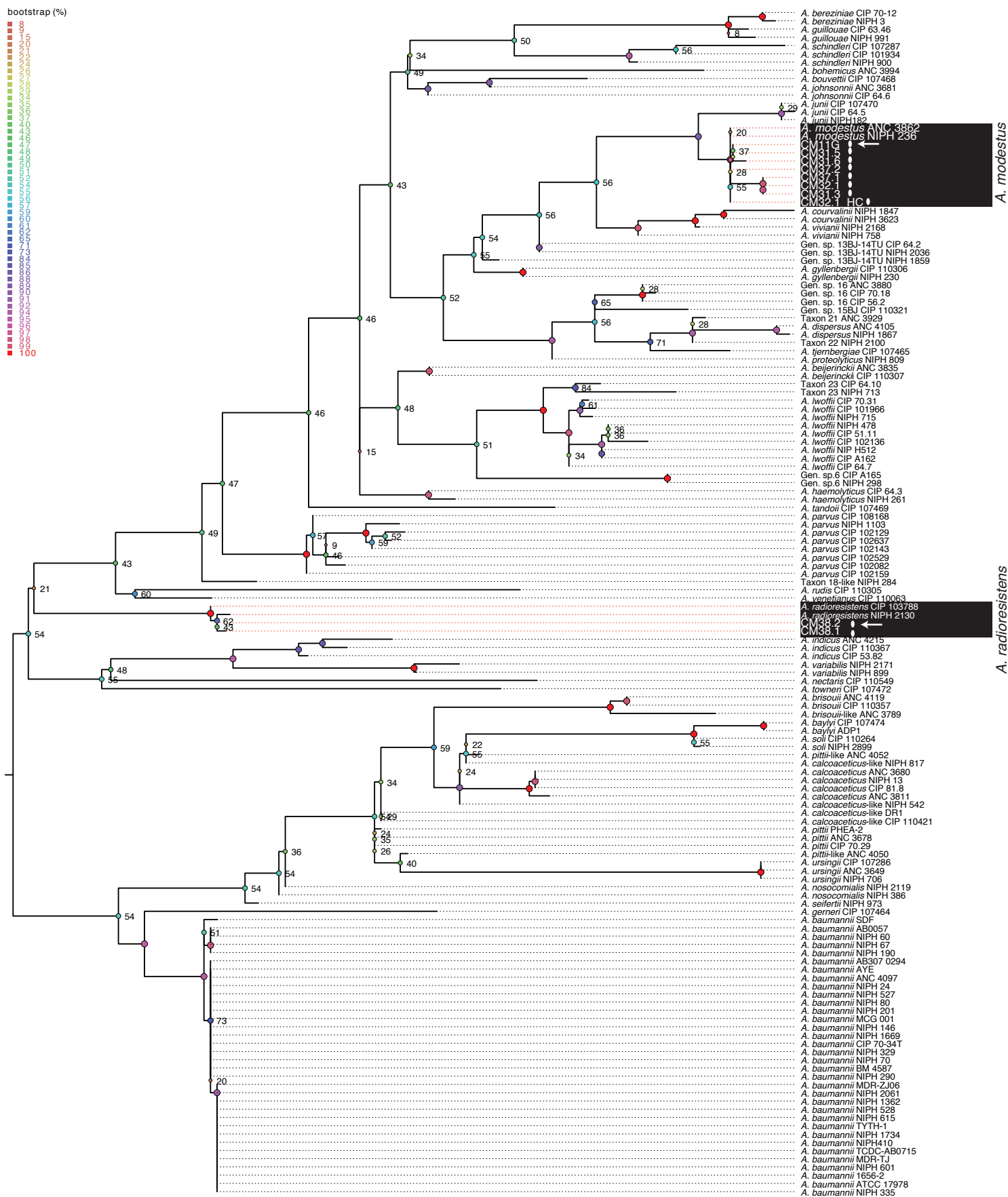

Supplement: Supplementary file 3 — Complete phylogenetic tree of Acinetobacter strains based on 16S rRNA gene sequences. The scale bar represents the average number of substitutions per site. (PDF 586 kb) [file 12864_2017_3925_MOESM3_ESM.pdf]

bootstrap (%)

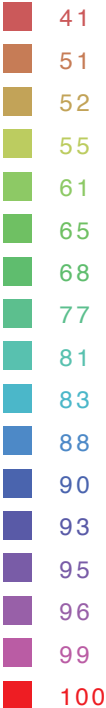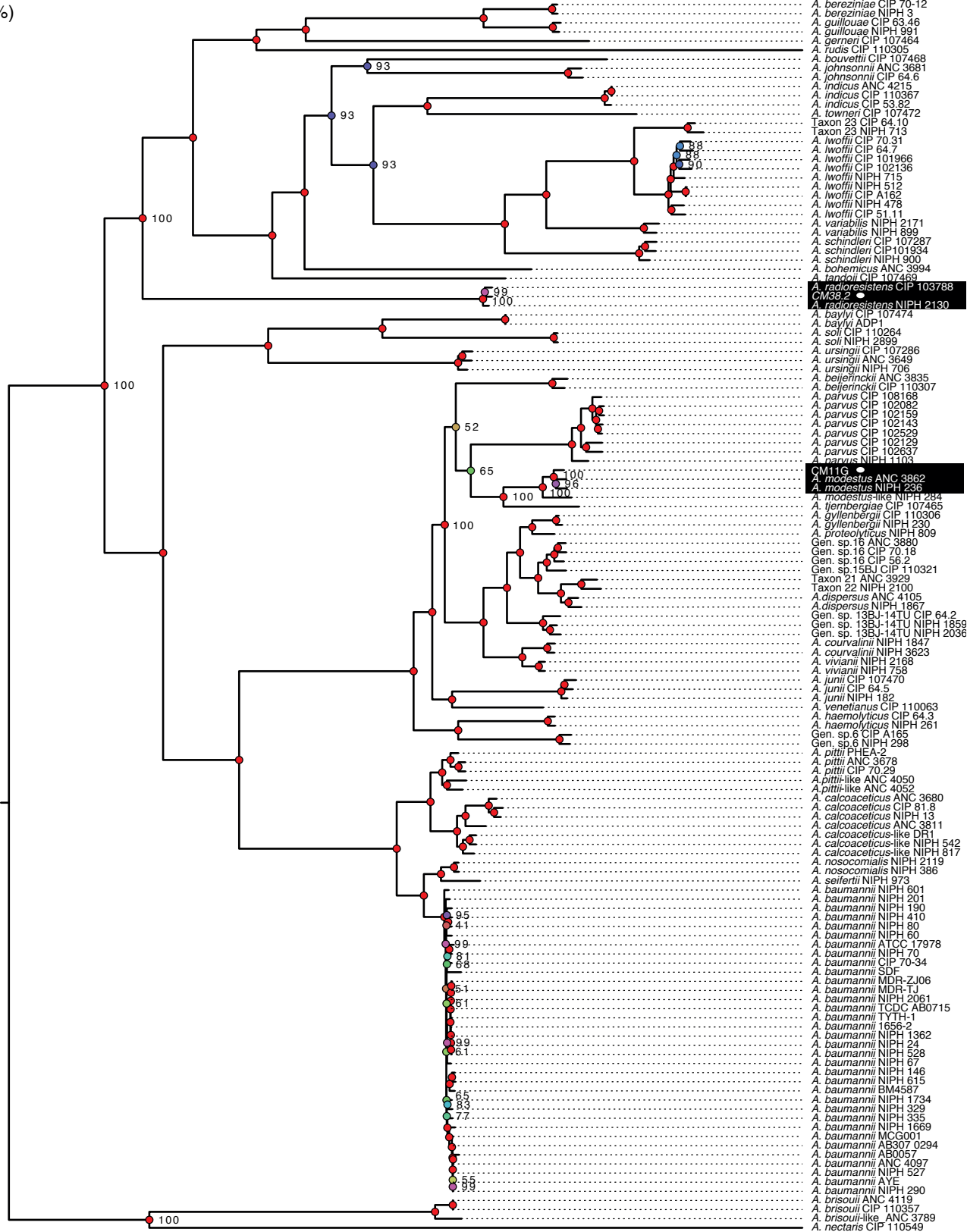

0.07

Supplement: Supplementary file 5 — Complete phylogenetic tree of the Acinetobacter genus based on the alignment of the protein families of the core-genome. The scale bar represents the average number of substitutions per site. (PDF 302 kb) [file 12864_2017_3925_MOESM5_ESM.pdf]

**A**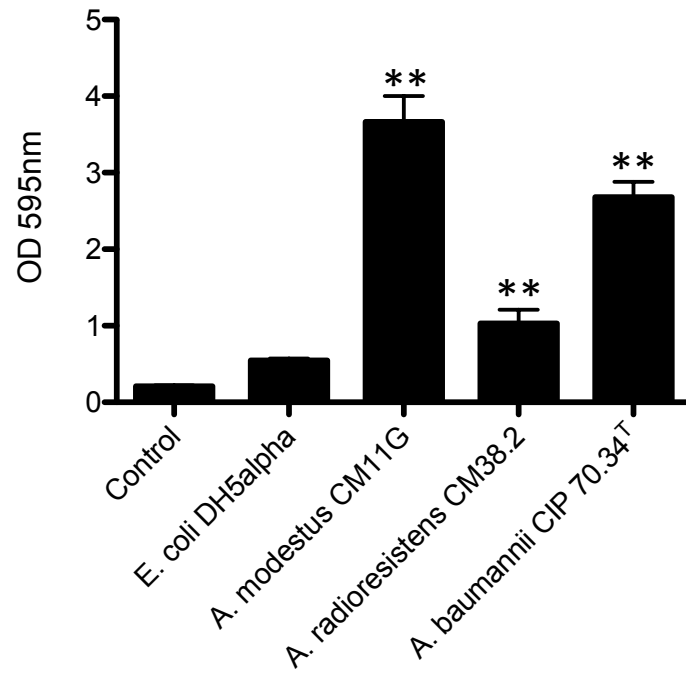**B**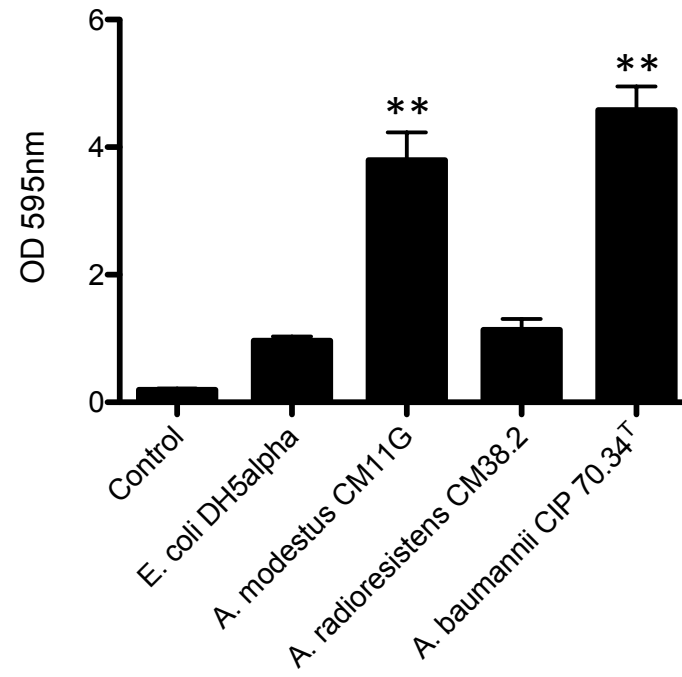

Supplement: Supplementary file 8 — Quantification of biofilm formation. Bacteria were incubated at 37 °C in Trypticase-Soy broth in polystyrene plate for 24H (A) or 48H (B). Data are expressed as mean ± Standard deviation, n = 6 in each group. **, P < 0.001 versus biofilm formation by Escherichia coli DH5a. Control: uninoculated wells. (PDF 56 kb) [file 12864_2017_3925_MOESM8_ESM.pdf]
