# Supplementary material for: Comparative genomic analysis of Acinetobacter strains isolated from murine colonic crypts
Source: BMC Genomics. 2017 Jul 11;18:525. doi: 10.1186/s12864-017-3925-x (PMC5505149; doi:10.1186/s12864-017-3925-x)
Supplement: Supplementary file 4 — Distribution of the genes of A. modestus CM11G and A. radioresistens CM38.2 following the functional categories obtained following RAST annotations. (DOCX 73 kb) [file 12864_2017_3925_MOESM4_ESM.docx]

| **Functional category** | ***A. modestus* CM11G** | | ***A. radioresistens* CM38.2** | |
| --- | --- | --- | --- | --- |
|  | Number | % | Number | % |
| Cofactors. Vitamins. Prosthetic Groups. Pigments | 216 | 10.1 | 233 | 9.4 |
| Cell Wall and Capsule | 79 | 3.7 | 98 | 4 |
| Virulence. Disease and Defense | 114 | 5.3 | 64 | 2.6 |
| Potassium metabolism | 20 | 0.9 | 16 | 0.6 |
| Miscellaneous | 26 | 1.2 | 83 | 3.4 |
| Phages. Prophages. Transposable elements. Plasmids | 11 | 0.5 | 18 | 0.7 |
| Membrane Transport | 85 | 4 | 68 | 2.8 |
| Iron acquisition and metabolism | 10 | 0.5 | 29 | 1.2 |
| RNA Metabolism | 116 | 5.4 | 114 | 4.6 |
| Nucleosides and Nucleotides | 75 | 3.5 | 88 | 3.6 |
| Protein Metabolism | 243 | 11.3 | 247 | 10 |
| Cell Division and Cell Cycle | 33 | 1.5 | 33 | 1.3 |
| Regulation and Cell signaling | 61 | 2.8 | 62 | 2.5 |
| Secondary Metabolism | 4 | 0.2 | 30 | 1.2 |
| DNA Metabolism | 103 | 4.8 | 86 | 3.5 |
| Fatty Acids. Lipids. and Isoprenoids | 126 | 5.9 | 161 | 6.5 |
| Nitrogen Metabolism | 20 | 0.9 | 20 | 0.8 |
| Dormancy and Sporulation | 2 | 0.1 | 2 | 0.1 |
| Respiration | 80 | 3.7 | 86 | 3.5 |
| Stress Response | 109 | 5.1 | 98 | 4 |
| Metabolism of Aromatic Compounds | 48 | 2.2 | 114 | 4.6 |
| Amino Acids and Derivatives | 307 | 14.3 | 375 | 15.2 |
| Sulfur Metabolism | 49 | 2.3 | 50 | 2 |
| Phosphorus Metabolism | 30 | 1.4 | 34 | 1.4 |
| Carbohydrates | 180 | 8.4 | 263 | 10.6 |
