# Supplementary material for: Comparative genomic analysis of Acinetobacter strains isolated from murine colonic crypts
Source: BMC Genomics. 2017 Jul 11;18:525. doi: 10.1186/s12864-017-3925-x (PMC5505149; doi:10.1186/s12864-017-3925-x)
Supplement: Supplementary file 6 — Antibiotic resistance pattern of the 10 Acinetobacter isolates. S: sensitive; I: intermediate; R: resistant. This panel of 32 antimicrobials agents is usually tested for non-fermentative Gram-negative bacteria. (DOCX 106 kb) [file 12864_2017_3925_MOESM6_ESM.docx]

| **Family** | **Antibiotic** | **CM11G** | **CM31.3** | **CM31.5** | **CM31.6** | **CM32.1 HC** | **CM32.1** | **CM 37.1** | **CM37.2** | **CM38.1** | **CM38.2** |
| --- | --- | --- | --- | --- | --- | --- | --- | --- | --- | --- | --- |
| Aminoglycosides | Amikacin | S | S | S | S | S | S | S | S | S | S |
| Aminoglycosides | Gentamycin | S | S | S | S | S | S | S | S | S | S |
| Aminoglycosides | Isepamicin | S | S | S | S | S | S | S | S | S | S |
| Aminoglycosides | Kanamycin | S | S | S | S | S | S | S | S | S | S |
| Aminoglycosides | Netilmicin | S | S | S | S | S | S | S | S | S | S |
| Aminoglycosides | Spectinomycin | R | R | R | R | R | R | R | R | S | S |
| Aminoglycosides | Streptomycin | R | R | R | R | R | R | R | R | S | S |
| Aminoglycosides | Tobramycin | S | S | S | S | S | S | S | S | S | S |
| Amphenicol | Chloramphenicol | S | S | S | S | S | S | S | S | R | R |
| Beta-lactamases | Amoxicillin | S | S | S | S | S | S | S | S | S | S |
| Beta-lactamases | Amoxicillin + Clavulanic acid | S | S | S | S | S | S | S | S | S | S |
| Beta-lactamases | Aztreonam | I | I | I | I | I | I | S | I | S | S |
| Beta-lactamases | Piperacillin + Tazobactam | S | S | S | S | S | S | S | S | S | S |
| Carbapeneme | Imipeneme | I | I | I | I | I | I | S | S | I | I |
| Cephalosporin | Cefamandole | I | I | I | I | I | I | I | I | I | I |
| Cephalosporin | Cefoperazone | I | I | I | I | I | I | I | I | I | I |
| Cephalosporin | Cefoxitine | S | S | S | S | S | S | S | S | S | S |
| Cephalosporin | Latamoxef | R | R | R | R | R | R | R | R | I | I |
| Cephalosporin (beta-lactamines) | Ceftriaxone | I | I | I | I | I | I | S | S | S | S |
| Cephalosporin, beta-lactamase | Ceftazidine | S | S | S | S | S | S | S | S | S | S |
| Cyclins | Tetracyclin | S | S | S | S | S | S | S | S | S | S |
| Fluoroquinolone | Ciprofloxacin | S | S | S | S | S | S | S | S | S | S |
| Fluoroquinolone | Ofloxacine | S | S | S | S | S | S | S | S | S | S |
| Macrocycle | Azithromycin | S | S | S | S | S | S | S | S | S | S |
| Penicillin | Piperacillin | S | S | S | S | S | S | S | S | S | S |
| Penicillin | Ticarcillin | S | S | S | S | S | S | S | S | S | S |
| Penicillin | Ticarcillin + Clavulanic acid | S | S | S | S | S | S | S | S | S | S |
| Quinolones | Nalidixic acid | S | S | S | S | S | S | S | S | S | S |
| Sulfonamides | Sulfonamides | S | S | S | S | S | S | S | S | S | S |
|  | Trimethoprim | S | S | S | S | S | S | S | S | S | S |
|  | Trimethoprim + Sulfonamides | S | S | S | S | S | S | S | S | S | S |
